# Factors Influencing COVID-19 Vaccination Hesitancy and Booster Dose Adherence Among University Students: A Cross-Sectional Study in Crete, Greece

**DOI:** 10.3390/healthcare13101115

**Published:** 2025-05-11

**Authors:** Izolde Bouloukaki, Antonios Christodoulakis, Athina Patelarou, Konstantinos Giakoumidakis, Michail Zografakis-Sfakianakis, Evridiki Patelarou, Ioanna Tsiligianni

**Affiliations:** 1 Department of Social Medicine, School of Medicine, University of Crete, 71500 Heraklion, Greece; izolthi@gmail.com (I.B.); i.tsiligianni@uoc.gr (I.T.); 2 Department of Nursing, School of Health Sciences, Hellenic Mediterranean University, 71410 Heraklion, Greece; apatelarou@hmu.gr (A.P.); kongiakoumidakis@hmu.gr (K.G.); mzografakis@hmu.gr (M.Z.-S.); epatelarou@hmu.gr (E.P.); 3 Department of Primary Care and Population Health, Medical School, University of Nicosia, 2417 Nicosia, Cyprus

**Keywords:** vaccination, hesitancy, university students, booster dose, cross-sectional, Greece, COVID-19

## Abstract

**Background/Objectives:** Vaccination hesitancy remains a global challenge, especially after the COVID-19 pandemic. We aimed to assess the prevalence of hesitancy towards regular COVID-19 vaccinations, including booster doses (additional doses beyond the primary to sustain or improve immunity), among university students and its associated factors. **Methods:** We conducted a cross-sectional study involving 190 university students from Hellenic Mediterranean University in Crete, Greece. Data were collected through an electronic survey distributed from July to October 2024. The survey included socio-demographic characteristics, health status factors, prior COVID-19 infection and vaccination history (general and for COVID-19), attitudes about COVID-19 vaccination, and the Attitude Towards Adult Vaccination (ATAVAC) scale. Qualitative data were also collected by addressing themes regarding ways to overcome vaccine hesitancy. **Results:** The study found that 64% of participants expressed hesitancy towards receiving COVID-19 booster doses. Factors contributing to this hesitancy were female gender, current smoking, pregnancy, concerns about vaccine side effects, a lack of confidence in vaccine efficacy, COVID-19 infection history, low perceived susceptibility to infection, and reliance on media information. Additionally, increased trust in the value of adult vaccination, adhering to recommendations of treating physician/scientist opinions, and prior adherence to vaccination were positively associated with regular COVID-19 vaccinations. A thematic analysis of the qualitative data identified four key strategies to overcome student vaccine hesitancy: enhancing health literacy, validating vaccine safety through further research, alleviating pandemic-related fears, and addressing distrust in authorities and opposition to mandatory vaccination. **Conclusions:** Our findings provide insights into the intricate factors and barriers of COVID-19 vaccination hesitancy among university students, thus emphasizing the need for more targeted interventions.

## 1. Introduction

Vaccination has always played a crucial role in global public health, effectively reducing the impact of numerous diseases like diphtheria, tetanus, pertussis, influenza, and measles [1]. The World Health Organization (WHO) estimates that between 2 and 3 million annual deaths could be prevented worldwide through widespread, effective vaccination [2]. Despite the availability of vaccines, vaccination hesitancy—defined as the reluctance or refusal to receive vaccines—poses a significant challenge and is listed among the WHO’s top ten threats to global health [3,4,5]. Factors such as trust in authorities, perceived efficacy, safety concerns, and misinformation drive hesitancy globally [6,7], with rates estimated at 32% across demographics, including university students [8,9,10]. Moreover, prior research has emphasized the role of cultural beliefs and misinformation in fostering vaccine hesitancy [11].

University students constitute an important demographic group that has the potential to significantly contribute to the reduction of vaccine-preventable diseases [3,10,12,13]. This is because they are particularly susceptible to contacting and transmitting infectious diseases due to their frequent interactions and mobility in crowded places, such as lecture halls, dormitories, and social events/gatherings [3,10,13,14]. Particularly during the COVID-19 pandemic, mandatory policies and the rapid deployment of vaccines raised ethical concerns about coercion versus collective responsibility, particularly among young adults, such as university students whose hesitancy may also stem from autonomy concerns [15]. Therefore, it is crucial to explore and comprehend the perspectives of university students regarding vaccine hesitancy and the barriers associated with it, as they also represent the future workforce [16,17,18]. Furthermore, their insights could be valuable in identifying potential trends and better understanding how attitudes towards vaccination are influenced by various factors, including age, influenza vaccination status, and previous COVID-19 infection [17,18]. This approach could facilitate the development of early educational interventions that may have a positive impact on vaccination behaviors in the long term globally.

In Greece, although 76.2% of population have received at least one COVID-19 vaccine dose [19], a significant portion remains unvaccinated as regard to booster doses, primarily due to societal polarization surrounding vaccines [20]. Moreover, Greece has faced numerous economic, political, and social challenges that could also influence attitudes towards vaccination [21,22]. The economic crisis, characterized by austerity measures, reduced healthcare access, and public trust in institutions, which may have indirectly influenced vaccination hesitancy [23,24]. Notably, COVID-19 vaccines were provided free of charge in Greece, potentially reducing financial barriers but not fully alleviating trust-related concerns [23,24]. Although previous studies have shown low levels of COVID-19 vaccination acceptance among the Greek general population and identified the aforementioned potential contributing factors [25,26,27], data specifically on university students remain limited [22]. Exploring the vaccine hesitancy for COVID-19 vaccines among university students in Greece could provide valuable insights to reduce vaccination hesitancy for other vaccines as well. We hypothesized that hesitancy is influenced by concerns over safety and efficacy, media reliance, and prior vaccination experiences, with trust in healthcare professionals mitigating hesitancy. Consequently, this study aimed to assess the prevalence of hesitancy toward regular COVID-19 vaccinations among university students in Crete, Greece. Moreover, our study also aimed to identify the potentially associated health and attitudinal factors and explore strategies to enhance vaccine acceptance.

## 2. Materials and Methods

### 2.1. Design and Sample

A cross-sectional study design was employed to investigate the attitudes, perceptions, and hesitancy toward COVID-19 vaccine and vaccines in general among university students enrolled at the Hellenic Mediterranean University (HMU) in Crete, Greece. The target population encompassed all registered undergraduate students at HMU. Inclusion criteria were as follows: (1) registered undergraduate students at HMU with active email accounts and (2) provision of informed consent. Exclusion criteria included students who did not complete all questionnaires. Given the exploratory nature of the study, a formal power calculation was not undertaken; nevertheless, the sample of 190 participants was deemed adequate to discern significant variations in the prevalence of hesitancy, as other similar studies have comparable sample sizes [28,29,30,31]. Consequently, all registered university students were invited to participate via direct email invitations sent to the student HMU mailing list from the university secretary to their student mailboxes. From all registered students, only 190 met the inclusion and exclusion criteria and were included in the study.

### 2.2. Data Collection

Data collection was conducted from July to October 2024. Participants were invited to complete the questionnaires, which were digitized in the Google Forms platform via an electronic link shared through university communication channels, including academic emails and student portals (eClass). Two weeks after the initial invitation, students received a kind reminder through the same channels. Participation was anonymous, voluntary, and informed consent was obtained electronically prior to participants’ access to the questionnaires.

### 2.3. Study Tools and Outcomes

The questionnaire was administered in Greek, and all participants, as students at a Greek university, were presumed to possess the ability to read and comprehend the Greek language. The study questionnaire (Supplementary Materials) consisted of three components. The first component was a self-administered questionnaire developed by the research team [27,32]. This questionnaire had 20 items related to (1) socio-demographic characteristics (age, gender, marital status, and years of education); (2) health status factors (smoking status, comorbidities, self-rated health status, and vulnerability status and family members’ vulnerability); (3) prior exposure or infection with COVID-19 and vaccination history (doses and side effects); (4) attitudes and beliefs about COVID-19 vaccination (items with questions of agreement or disagreement); and (4) level of information and questions about the degree of influence of parameters related to vaccine uptake (religion, politics, science, media and internet, and anti-vaccine movement) and whether they were vaccinated for influenza during the last season. It should be noted that the age groups were categorized into 17–20, 21–24, and ≥25 years to align with typical university student stages: early undergraduate, mid-to-late undergraduate, and mature students, respectively. Self-rated health was classified as good/excellent, fair, or poor, consistent with standard health survey practices to gauge subjective health perceptions.

The second component evaluated the attitudes and hesitancy toward vaccination utilizing the Attitude Towards Adult Vaccination (ATAVAC) scale, which was originally developed and validated in Greek [33]. The ATAVAC scale is designed to assess attitudes and perceptions toward adult vaccination, including hesitancy and associated barriers. ATAVAC is a valuable tool for identifying determinants of vaccine hesitancy and has been demonstrated to correlate strongly with vaccination behavior, making it a valuable resource for public health research and intervention design [22,34,35]. The ATAVAC is composed of 11 items divided into three subscales: the value of adult vaccination (7 items), safety concerns (2 items), and perceived barriers (2 items). Respondents rate their agreement on a six-point Likert scale, from 1 (strongly disagree) to 6 (strongly agree). The final score resulted from the sum of eleven items’ scores (with seven reverse-scored items) divided by eleven. The total score ranged from 11 to 66, with higher scores indicating more favorable attitudes toward vaccination. Increased scores on the “Value of adult vaccination” subscale corresponded to more favorable views of adult immunization. Conversely, higher scores on the “Safety concerns” and “Perceived barriers” subscales indicated fewer concerns about vaccine safety and fewer practical barriers to vaccination. The ATAVAC scale was chosen for its proven reliability (Cronbach’s alpha = 0.821) and relevance to adult vaccination attitudes [22,33,34].

In the third part, participants were prompted to provide detailed explanations of their suggestions for improving COVID-19 vaccine confidence via an open-ended question. The collected data underwent independent analysis by two qualitative data analysis experts. The authors used an open coding approach to analyze the data, collaboratively developing and refining a coding framework based on recurring patterns for all participant responses. Codes were refined through consensus, and four key themes were defined when saturation was reached—i.e., no new concepts emerged—following Braun and Clarke’s thematic analysis guidelines [36].

### 2.4. Statistical Analysis

Data analysis was performed using SPSS version 25 (SPSS Inc., Chicago, IL, USA). All continuous variables were tested for normality using several methods (skewness and kurtosis, the proximity of the mean to the median, visual inspection of their histograms, Q-Q plots, and box plots). Normally distributed continuous variables were reported as their mean ± standard deviation. Non-normally distributed variables were reported as their median (interquartile range, IQR: 25th–75th percentile, representing the middle 50% of the data). Qualitative variables were represented using absolute numbers or percentages. Independent samples *t*-tests (two-tailed, assuming normality) or Mann–Whitney U tests (for non-normal data) were employed to compare continuous variables between hesitant and non-hesitant groups. For categorical variables, we utilized Pearson’s chi-square test. We used multivariate logistic regression to identify factors affecting COVID-19 vaccination hesitancy. To account for potential bias, the analysis was adjusted for age, gender, smoking status, education level, and co-morbidities. Calculations were performed to derive the odds ratios (ORs) and their respective 95% confidence intervals (95% CIs). We used collinearity statistics to check for multicollinearity among the predictor variables. The model showed acceptable collinearity, as indicated by the tolerance and variance inflation factor. Statistical significance was determined by a *p*-value threshold of <0.05.

## 3. Results

### 3.1. Study Population Characteristics

The study sample consisted of 190 participants, comprising 32 males (17%) and 158 females (83%). The majority of participants were under the age of 25, with 100 (53%) aged between 17 and 20, 61 (32%) aged between 21 and 24, and 29 (15%) aged 25 and above. The second-year student group represented the largest proportion (56%). In terms of chronic diseases, 29 participants (15%) had at least one condition. These conditions included thyroid disease (7%), inflammatory arthritis (3%), diabetes mellitus (2%), asthma (2%), arrhythmias (1%), ulcerative colitis (1%), and multiple sclerosis (1%). The majority of participants (72%) rated their health as good or excellent, while 26% rated it as fair and 2% rated it as poor. Further details regarding the hesitant and non-hesitant groups are provided in Table 1.

### 3.2. Coverage and Hesitancy of Regular COVID-19 Vaccination

Overall, 145 out of 190 subjects (76%) had received the COVID-19 vaccine (at least one dose). The majority, however, received their last dose two (46%) or three (46%) years prior, with only 8% having their last dose within the past year. Mild side effects were reported by 48% of vaccinated participants, while 14% reported moderate-to-severe side effects. Moreover, a significant portion of those vaccinated (57%) reported that their decision was guided by their personal views on the COVID-19 vaccine. Nevertheless, a large proportion of respondents also highlighted the importance of several other factors, including fear of infection (41%), professional limitations (35%), physician recommendations (27%), and the availability of free vaccines (11%).

In total, 64% of the individuals participating in the study expressed hesitancy to-wards receiving the COVID-19 vaccine on a regular basis. Hesitancy rates were similar among health science students (62%) and students at other universities (73%; *p* = 0.277). The baseline characteristics of the study population, according to hesitancy, are shown in Table 1. Hesitant participants were mostly females (88 vs. 75%, *p* = 0.027) compared to non-hesitant. The two groups did not differ significantly in terms of age, area or level of education, marital status, smoking status, chronic disease, and self-rated health status.

Sources of information contributing to vaccine acceptance were primarily doctor/scientist opinion (92%), followed by media/internet (13%), with smaller influences from anti-vaccination campaigns, government trust, and religious beliefs (each at 4%).

A comparison of experiences, attitudes, and beliefs regarding COVID-19 and vaccination is shown in Table 2 and Table 3 for those hesitant versus those not hesitant about COVID-19 regular vaccination. Higher COVID-19 vaccination doses and prior influenza vaccination were associated with less hesitancy towards COVID-19 vaccination.

On the other hand, participants expressing concerns about vaccine side effects, observing such effects in their social circles, questioning vaccine efficacy, and believing natural infection provided superior immunity demonstrated higher rates of vaccine hesitancy (Table 3).

### 3.3. Attitudes Towards Adult Vaccination

A substantial percentage (57%) of the participants indicated that the COVID-19 pandemic did not affect their beliefs and attitudes regarding vaccination in general; 31% reported a positive impact, while only 12% reported a negative one. Participants’ mean score on the ATAVAC scale was 4.64 ± 0.64 (min. 2.55–max. 7.36). Their subscale scores were as follows: (a) value of adult vaccination 4.86 ± 0.67 (min. 2.0–max. 6.0); (b) safety concerns 3.24 ± 1.36 (min. 1.0–max. 6.0); and (c) perceived barriers 4.88 ± 1.09 (min. 1.0–max. 6.0). Scores on the ATAVAC scale and respective subscales and items according to participant’s COVID-19 vaccine hesitancy are illustrated in Table 4. Specifically, participants who expressed COVID-19 vaccine hesitancy had significantly lower scores across the ATAVAC scale (lower vaccine hesitancy in general)—particularly on the subscales assessing the value and safety of adult vaccination—and on eight out of eleven individual items.

### 3.4. Predictors of Vaccine Hesitancy

After controlling for confounding variables, the multivariate logistic regression analysis (Table 5) showed that females were 2.6 times more likely than men to report hesitancy [OR: 2.645 (1.180–5.929), *p* = 0.018]. Current smokers were also more likely to be hesitant [OR: 2.359 (1.080–5.153), *p* = 0.031]. Previous COVID-19 vaccination with ≥3 doses [OR: 0.136 (0.054–0.339), *p* < 0.001] and previous flu vaccination [OR: 0.439 (0.212–0.910), *p* = 0.027] had a negative correlation with reporting hesitancy.

Participants who expressed concerns about vaccine side effects either for themselves [OR: 2.135 (1.126–4.049), *p* = 0.020] or their family and friends [OR: 3.079 (1.177–8.056), *p* = 0.022] and had low confidence in the vaccine’s effectiveness [OR: 12.132 (3.527–41.734), *p* < 0.001] were more inclined to exhibit hesitancy. Furthermore, a history of COVID-19 infection [OR: 3.393 (1.047–11.002), *p* = 0.042], a belief in superior immunity from natural infection compared to vaccination [OR: 9.558 (1.190–76.742), *p* = 0.034], and a perception of low risk or mild infection severity [OR: 9.368 (1.099–79.877), *p* = 0.041] were all associated with increased vaccine hesitancy. Pregnancy also significantly contributed to vaccine hesitancy [OR: 9.172 (0.037–0.793), *p* = 0.024].

On the contrary, a recommendation from treating physicians [OR: 0.108 (0.013–0.870), *p* = 0.037], higher ATAVAC value scores [OR: 0.216 (0.099–0.472), *p* <0.001], increased trust in the value of adult vaccination (ATAVAC subscale score OR: 0.137 (0.058–0.321), *p* < 0.001), and fewer concerns about the safety of vaccines (ATAVAC subscale score OR: 0.615 (0.475–0.796), *p* < 0.001) were linked to less hesitancy, whereas media/internet information was associated with higher odds of hesitancy [OR: 6.218 (1.692–22.848), *p* = 0.006].

### 3.5. Participants Perspectives in Addressing Vaccine Hesitancy

Regarding strategies in addressing vaccine hesitancy, four main themes emerged from the analysis of participant’s responses to the open-ended question: (a) enhancing health literacy; (b) validating vaccine safety through further research; (c) alleviating pandemic-related fears; and (d) addressing distrust in authorities and opposition to mandatory vaccination. Example quotes per theme are presented in Table 6.

## 4. Discussion

In this cross-sectional study, we found that a significant proportion (64%) of participants expressed hesitancy towards receiving booster doses of the COVID-19 vaccine. Our study also identified several factors that could contribute to this hesitancy, including female gender, current smoking status, pregnancy, concerns about vaccine side effects, a lack of confidence in vaccine efficacy, COVID-19 infection history, perceived low susceptibility to COVID-19 infection (particularly the severe form), and reliance on media information. In addition, we found a positive association between increased trust in the value of adult vaccination and adhering to the recommendations of the treating physician and scientist with having previously received the influenza vaccine or having received more than three COVID-19 vaccine doses. Furthermore, qualitative data identified four key themes/recommendations to address student vaccine hesitancy and enhance acceptance, including improving health literacy regarding vaccination, conducting further research to validate vaccine safety, alleviating pandemic-related anxieties, and addressing distrust in authorities and opposition to mandatory vaccination. Consequently, this study offers critical insights into COVID-19 vaccination hesitancy among university students in Greece, a part of the population that holds significant importance for public health strategies due to their frequent social interactions and future roles, particularly as healthcare professionals. Comprehending vaccine hesitancy within this group is paramount amidst global challenges, as acknowledged by the World Health Organization, which identifies hesitancy among the top ten threats to health. Our findings contribute to the broader literature by underscoring specific factors that influence hesitancy in a post-economic crisis context.

Overall, the level of vaccination hesitancy was relatively high in this study, with 64% of participants grouped in the hesitant group. The percentage of students hesitant to receive regular COVID-19 vaccines seems to differ widely across the globe; some regions reported rates between 38% and 74% [18,37,38,39], while others reported rates as low as 18% to 27% [40,41]. For example, vaccination hesitancy was 27% in China [40], 52% in Pakistan [38], and 29.2% in Finland [18]. Existing research on Greek university students’ vaccine hesitancy is limited to a single study (from another region of Greece than this study), which evaluated overall vaccine acceptance and found that 75% of participants demonstrated some degree of hesitancy [22]. A potential explanation for this discrepancy could be attributed to the varying socioeconomic circumstances between countries and the diverse participant demographics across studies [40,41].

An interesting finding of the present study was that vaccine hesitancy rates were similar for both health science and non-health science students. Specifically, vaccine hesitancy among health science students reached 62%, a figure higher than previous international studies [37,39,41]. A systematic review conducted across multiple countries (including the United States, Saudi Arabia, Kazakhstan, Brazil, Italy, France, Poland, South Korea, India, China, Turkey, and others) globally has indicated that student vaccination hesitancy ranges between 38% and 44% [39]. This range is significantly lower than the value observed in our study (62%). Meanwhile, a meta-analysis of studies that focused on dental students suggested that they have 39.5% vaccination hesitancy [37]. Moreover, a meta-analysis conducted among healthcare students highlighted that 31.2% of these students exhibited vaccination hesitancy [41].

Our study highlights four key factors contributing to vaccine hesitancy. More specifically, the main reasons for vaccination hesitancy among university students were concerns about potential side effects, skepticism regarding vaccine efficacy, a history of COVID-19 infection, a belief that they are not at high risk of severe disease, and reliance on media information. This aligns with another study, which found that United Arab Emirates university students similarly doubted vaccine efficacy, especially concerning their rapid development [42]. Our findings suggest that a lack of faith in how well vaccines work is the most important factor contributing to participants being hesitant about getting vaccinated. Ranking second were the belief in naturally acquired immunity’s superiority over vaccine-induced immunity and the perception of low personal risk from severe infection. A possible explanation for this could be that some students believe their age and health status render them less susceptible to severe COVID-19 [43]. This belief may be explained due to better health status at a young age leading to a lack of urgency about getting vaccinated.

Our study also identified the perceived lack of vaccine safety as another significant factor influencing hesitancy. While the rapid development and deployment of COVID-19 vaccines initially raised safety and efficacy concerns as one could expect [44,45], these issues still persist. Furthermore, prior studies indicate that the degree of skepticism towards vaccine safety amongst students is attributed to misinformation and to a perceived low personal risk of infection, and this finding is consistent, even within healthcare student populations [46,47,48,49]. On the other hand, our findings suggest that recommendations from treating physicians and scientists’ opinions were the primary source of information for vaccine acceptance, thus influencing participants’ vaccination hesitancy. Our previous study also supports this finding, underscoring healthcare professionals’ and scientist’s crucial role in promoting vaccination acceptance [32]. Consequently, vaccination hesitancy presents a multifaceted challenge even four years after the COVID-19 pandemic, with the decision-making process being intricate and subject to numerous influences. Studies have suggested that the rise in vaccine hesitancy during the pandemic was significantly influenced by the spread of misinformation, public distrust in government and political decision-making, doubts about vaccine safety and efficacy, and an underestimation of individual risk [5,50].

Our findings suggest that the influence of media and the internet was linked to a six-times greater risk of vaccine hesitancy. This finding concurs with previous research suggesting that students’ attitudes towards vaccines, particularly COVID-19 vaccines, are substantially influenced by social media misinformation [51]. This finding could be explained due to the fact that students’ attitudes towards vaccines, including COVID-19 vaccines, are significantly shaped by access to social media information [52]. Meanwhile, we found that pregnancy was positively associated with vaccine hesitancy. This finding aligns with a mixed-method analysis study that further suggested safety concerns, healthcare professionals advice (waiting until second trimester or until after birth/breastfeeding), taking other precautions, misinformation, and complacency (not worried about getting COVID-19) as the primary reasons for this hesitancy [53].

A notable finding of the present study was the high level of confidence in general adult vaccination reported by study participants, as depicted by the high ATAVAC scores. However, participants reporting hesitancy toward COVID-19 vaccination also had lower ATAVAC scores (lower general vaccination hesitancy), especially as regards the value of adult vaccination and the safety concern subscales, which reflect disbelief, confirming similar findings from previous studies examining predictors of COVID-19 vaccine uptake in the general adult population [35] and medical and biomedical science students [22]. Participants’ vaccination decisions regarding the COVID-19 vaccine were positively associated with their ATAVAC scores, supporting the scale’s use in identifying vaccine hesitancy. The implications of this finding are substantial, as elevated COVID-19 vaccine hesitancy could negatively influence vaccination rates for other types of vaccines. Consequently, healthcare policy makers and professionals should consider this when promoting all types of vaccines.

Our study has also identified various key factors that could influence vaccine acceptance. These include enhancing health literacy, establishing trust in vaccine safety through research, addressing pandemic-related anxieties, and easing concerns about authority and mandatory vaccination. Improving health literacy is critical, involving educating the public about vaccine mechanisms and benefits and dispelling myths [54,55,56]. This could be achieved by creating an integrated service model that brings together government agencies, healthcare institutions, and the media to ensure information meets public needs [57]. Building trust in vaccine safety requires ongoing research and transparent communication about vaccine development, efficacy, and potential side effects [58,59,60,61]. For example, sharing research findings regularly could improve public confidence [58]. Addressing pandemic-related anxieties is also crucial; the COVID-19 pandemic has heightened fears about health risks, economic stability, and social disruption [62,63,64]. Therefore, public health messages could be more empathetic, addressing these concerns while explaining how vaccines could help restore normalcy [62,63,64]. Finally, addressing concerns about authority and mandatory vaccination is essential. Health authorities could more actively engage with communities, explaining the rationale behind vaccination policies and fostering open dialogue rather than imposing mandates [6,62,63,64]. The aforementioned suggestions could be vital for improving vaccine acceptance and strengthening public trust in the COVID-19 vaccines and vaccines in general.

Our findings suggest that Greek university students have high levels of COVID-19 and general vaccination hesitancy, which has important implications for public health and primary care. First, this reluctance could potentially shape their future attitudes towards health interventions, impacting their participation in preventive health measures. Therefore, it is crucial to address the underlying reasons for vaccine hesitancy in order to develop targeted public health strategies that could improve vaccination rates during outbreaks and for regular immunization schedules. By implementing educational initiatives and creating supportive environments, universities can actively promote vaccine uptake, thereby contributing to public health efforts in managing future disease outbreaks. Healthcare policymakers could also play a pivotal role in educating and informing the younger generation about the importance of health awareness [3,10,13,14]. This approach would not only benefit local communities but may also have broader global impacts, as students interact with international peers, leading to improved global public health outcomes and the development of a resilient, health-conscious global community [3,10,13,14]. Building on our findings, future research could integrate longitudinal, comparative, and even interventional studies to better understand vaccine hesitancy among university students, thus helping in the development of more targeted interventions to improve vaccination acceptance in these students. Longitudinal studies could examine how hesitancy changes over time, identifying key moments for an intervention. Moreover, future studies could evaluate tailored interventions that focus on reducing vaccine hesitancy in university students, such as peer campaigns and/or digital tools [17]. Social media’s role in spreading misinformation could also be explored and test interventions to mitigate this spread, while qualitative studies on psychological factors (risk perception, cultural mistrust) could help develop more targeted behavioral interventions. Finally, examining the effect of health equity and policy impacts (like vaccine mandates) could ensure more inclusive and effective strategies, creating a cohesive research agenda addressing global and local health issues.

### Limitations

Our study has a few notable strengths; first, it centers on a critical demographic group—university students—utilizing a mixed-method approach. Second, the study’s setting in Crete, Greece, during the post-economic crisis offers unique contextual insights into the factors that contribute to hesitancy. Nevertheless, despite these valuable insights into the hesitancy of Greek university students towards COVID-19 vaccination, it is important to acknowledge certain limitations. First, this was a single-centered study, and the sample mainly consisted of individuals with health science backgrounds, so it may not fully represent the wider university student population of South Greece. Second, the sample was predominantly female (83%) and from health science backgrounds (88%), introducing potential selection bias that may limit the generalizability of the findings to male students or other disciplines. Third, the cross-sectional design of the study restricts the ability to establish definitive cause-and-effect relationships between the identified factors and vaccine hesitancy. Fourth, since acceptance and hesitancy towards vaccines can change over time due to new evidence and evolving public perceptions, the results of this study may not fully capture future trends. Thus, it is necessary to conduct longitudinal studies to examine how attitudes and hesitancy evolve over time. Future research could include a more diverse and representative sample in order to strengthen the reliability and applicability of the study’s conclusions.

## 5. Conclusions

In conclusion, our study findings suggest that Greek university students have high COVID-19 vaccination hesitancy, driven by safety concerns, efficacy doubts, and media influence. Our findings further suggest that addressing this hesitancy requires enhancing health literacy, ensuring transparent research, countering misinformation, and leveraging trusted medical professionals. By effectively addressing vaccine hesitancy, healthcare policymakers could not only increase COVID-19 vaccination rates among students but also contribute to better preparedness for future public health challenges.

## Figures and Tables

**Table 1:** Characteristics of all the 190 participants categorized based on COVID-19 vaccination hesitancy.

Characteristics	Non Hesitant (*n* = 69)	Hesitant (*n* = 121)	*p*-Value
**Demographics**			
**Gender, males**	17 (25%)	15 (12%)	0.027
**Age Groups (years)**			
17–20	34 (49%)	55 (55%)	0.442
21–24	26 (38%)	35 (29%)	
≥25	9 (13%)	20 (17%)	
**Area of study**			
Health Sciences (Nursing, Nutrition and Dietetics, Social Work)	63 (91%)	104 (86%)	0.277
Others (Agricultural, Management and Economics, Engineering, Music and Optoacoustic Technologies)	6 (9%)	17 (14%)	
**Year of study**	3 (1, 4)	2 (1, 4)	0.047
First or second	45 (49%)	72 (60%)	0.172
Third or above	35 (51%)	49 (41%)	
**Married/Partner**	3 (5%)	15 (14%)	0.052
**Smoking status**			
Current	12 (17%)	36 (30%)	0.054
Never/Former smoker	57 (83%)	85 (70%)	
**Chronic health condition (≥1)**	9 (13%)	20 (17%)	0.521
**Self-rated health**			
Good/Excellent	55 (80%)	81 (67%)	0.119
Fair	14 (20%)	40 (33%)	
Bad	0 (0%)	4 (3%)	
**Self-identified as vulnerable group**	7 (10%)	11 (9%)	0.811
**Living with vulnerable individuals**	24 (35%)	33 (27%)	0.277

**Table 2:** Reported experiences with COVID-19 virus and vaccination for individuals who were hesitant and non-hesitant towards COVID-19 booster vaccination.

Experiences	Non Hesitant (*n* = 69)	Hesitant (*n* = 121)	*p*-Value
**Previous COVID-19 infection, N (%)**			
No infection	13 (18%)	15 (12%)	0.169
Asymptomatic or mild symptoms	30 (44%)	44 (36%)	
Moderate or severe symptoms	26 (38%)	62 (51%)	
**COVID-19 vaccination doses**			
Zero	7 (10%)	38 (31%)	<0.001
One	3 (4%)	5 (4%)	
Two	26 (38%)	54 (45%)	
Three	32 (46%)	23 (19%)	
≥4	1 (1%)	1 (1%)	
**COVID-19 vaccine Side-effects (*n* = 145)**			
None	26 (42%)	29 (35%)	0.409
Mild	30 (48%)	40 (48%)	
Moderate/severe	6 (10%)	14 (17%)	
**Flu vaccination**			
Yes	51 (74%)	68 (56%)	0.015
No	18 (26%)	53 (44%)	

**Table 3:** Reported attitudes and beliefs about COVID-19 vaccination in groups that were hesitant and non-hesitant towards the COVID-19 booster vaccination.

Attitudes	Non Hesitant (*n* = 69)	Hesitant (*n* = 121)	*p*-Value
Fear of vaccine side effects	28 (41%)	75 (62%)	0.004
Reported vaccine side effects among family/friends	6 (9%)	31 (21%)	0.005
Low perceived efficacy of vaccine	3 (4%)	45 (37%)	<0.001
Early vaccine distribution	23 (33%)	57 (41%)	0.064
No need due to previous COVID-19 infection	4 (6%)	21 (17%)	0.023
Belief that infection confers much greater immunity than a vaccine	1 (1%)	15 (12%)	0.009
Perception of low susceptibility to disease or possible infection would not be severe	1 (1%)	10 (8%)	0.053
Against vaccinations in general	4 (4%)	6 (4%)	0.727
Information insufficiency	26 (38%)	45 (37%)	0.946
Belief that vaccine development is a way for pharmaceutical companies to make a profit/conspiracy belief	4 (6%)	15 (12%)	0.145
Pregnancy	6 (9%)	3 (3%)	0.052

**Table 4:** Mean scores of ATAVAC scale (total score), subscales, and items according to their COVID-19 vaccine hesitancy.

Mean Score per Category	Non Hesitant (*n* = 69)	Hesitant (*n* = 121)	*p*-Value
**ATAVAC scale (total score)**	4.93 ± 0.46	4.42 ± 0.66	<0.001
**Subscales**			
Value of adult vaccination subscale	5.21 ± 0.45	4.63 ± 0.70	<0.001
Safety concerns subscale	3.83 ± 1.19	2.91 ± 1.33	<0.001
Perceived barriers subscale	4.91 ± 0.94	4.86 ± 1.08	0.754
**Items**			
I fear the immediate complications of a vaccine (such as allergic reactions)	3.56 ± 1.29	2.94 ± 1.39	0.004
I fear the potential impact of vaccines on my health in the future	4.12 ± 1.44	2.88 ± 1.49	<0.001
I believe in the value of vaccination	5.65 ± 0.59	4.95 ± 0.99	<0.001
It is difficult for me to access the doctor for vaccination (I cannot find an appointment, or the office is too far away or there is no transportation, etc.)	4.93 ± 1.02	4.89 ± 1.13	0.776
I believe that vaccines are necessary for adults	5.21 ± 0.89	4.54 ± 1.17	<0.001
I believe that the benefits of vaccination outweigh the potential risks	5.43 ± 0.74	4.32 ± 1.14	<0.001
I think if I get ill, I will get more antibodies (better body auto-defense) than if I just get a vaccination	4.26 ± 1.37	3.50 ± 1.38	<0.001
I believe that vaccines are very effective in protecting me from getting a disease	5.01 ± 0.78	4.05 ± 1.23	<0.001
I haven’t had a vaccine as an adult so far, so I don’t need it	5.49 ± 0.66	5.10 ± 1.13	0.013
I believe that vaccines should only be given to children	5.38 ± 0.65	5.25 ± 0.59	0.151
I have financial difficulty in paying for a visit to a doctor or I can’t afford the transportation costs to the office to have the vaccines I need	4.83 ± 1.13	4.83 ± 1.28	0.975

**Table 5:** Multivariate logistic regression analysis of factors associated with COVID-19 vaccine hesitancy.

Variables	Adjusted OR (95% CI)	*p*-Value
**Socio-demographic factors**		
Females (vs. males)	2.645 (1.180–5.929)	0.018
Age groups		
17–20 (ref *) years	1	
21–24 years	0.601 (0.297–1.216)	0.157
≥25 years	1.212 (0.482–3.048)	0.683
Area of study [Health science vs. others (ref *)]	0.320 (0.099–1.033)	0.057
Year of study		
First or second (ref *)	1	
Third or above	0.504 (0.179–1.423)	0.196
Single vs. married (ref *)	3.073 (0.684–13.800)	0.143
Current vs. former/no smoking (ref *)	2.359 (1.080–5.153)	0.031
**Health status factors**		
Chronic health condition [(≥1 vs. o (ref *)]	0.885 (0.355–2.205)	0.793
Self-rated health good/excellent vs. fair/bad (ref *)	0.645 (0.288–1.445)	0.286
Self-rated as vulnerable group [vs no (ref *)]	0.407 (0.107–1.549)	0.188
Living with vulnerable individuals [vs no (ref *)]	0.667 (0.335–1.331)	0.251
**Experiences**		
Previous COVID-19 infection [moderate/severe vs. mild/none(ref *)]	1.718 (0.674–4.376)	0.257
COVID-19 vaccinationDoses [(≥3 doses vs. <3 doses (ref *)]	0.136 (0.054–0.339)	<0.001
COVID-19 vaccinationSide effects [moderate severe vs. none/mild(ref *)]	1.940 (0.624–6.031)	0.252
Flu vaccination [yes vs. no (ref *)]	0.439 (0.212–0.910)	0.027
Attitudes [yes vs. no (ref *)]		
Fear of vaccine side effects	2.135 (1.126–4.049)	0.020
Reported vaccine side effects among family/friends	3.079 (1.177–8.056)	0.022
Low perceived efficacy of vaccine	12.132 (3.527–41.734)	<0.001
Early vaccine distribution	1.718 (0.893–3.304)	0.105
No need due to previous COVID-19 infection	3.393 (1.047–11.002)	0.042
Belief that infection confers much greater immunity than a vaccine	9.558 (1.190–76.742)	0.034
Perception of low susceptibility to disease or possible infection would not be severe	9.368 (1.099–79.877)	0.041
Information insufficiency	0.942 (0.491–1.809)	0.858
Belief that vaccine development is a way for pharmaceutical companies to make a profit/conspiracy belief	2.520 (0.742–8.557)	0.138
Pregnancy	9.172 (0.037–10.793)	0.024
Sources of information [yes vs. no (ref *)]		
Anti-vaccination campaigns	3.365 (0.385–29.384)	0.272
Doctors’ recommendation/scientist opinion	0.108 (0.013–0.870)	0.037
Media/internet	6.218 (1.692–22.848)	0.006
Government trust	4.831 (0.494–47.223)	0.176
Religious beliefs	3.980 (0.455–34.849)	0.212
**ATAVAC scale (total score)**	0.216 (0.099–0.472)	<0.001
Subscales		
Value of adult vaccination subscale	0.137 (0.058–0.321)	<0.001
Safety concerns subscale	0.615 (0.475–0.796)	<0.001
Perceived barriers subscale	1.004 (0.725–1.392)	0.979

* The reference category against which comparisons are being conducted.

**Table 6:** Example quotes from the open-ended question exploring the enablers in addressing vaccine hesitancy, allocated in overarching themes. Participants’ sex, approximate age, and area of study are presented for each quote.

Themes	Example Quotes
Improving health literacy related to vaccination	“The media should prioritize accurate information, not spreading misinformation”. Male, ≥25 years old, Agricultural science. “Reliable data from scientists”. Male, 17–18 years old, Health science. “More details about the side effects”. Male 23–24 years old, Engineering.
Validating vaccine safety through further research	“We need more tests and research to make the vaccine better and avoid any unexpected side effects or bias”. Female, 19–20 years old, Health science. “If they had spent more time testing the vaccine, it probably would have been better”. Female, 19–20 years old, Health science. “Adequate time is required to assess side effects and determine which groups experience them”. Female, 19–20 years old, Health science. “Closely observe vaccinated populations to ensure the integrity of research data. Release of all research results. All scientific opinions deserve hearing; no voice should be suppressed”. Female, ≥25 years old, Agricultural science. “Further investigation is needed to explore the connection between the vaccine and the observed increase in both sudden deaths and cardiomyopathy among young people”. Male, 19–20 years old, Health science.
Alleviating pandemic-related fears	“A resurgence of severe COVID-19 cases and significant worsening of the situation”. Female, 19–20 years old, Health science. “Rapid increase in cases and deaths”. Female, 17–18 years old, Health science. “If COVID-19 virus was on the rise again as before, and cases were rising rapidly”. Female, 17–18 years old, Health science. “A new pandemic”. Female, 21–22 years old, Health science. “If COVID-19 was again on the rise and the experts recommended it [the vaccine]”. Female, 17–18 years old, Health science.
Addressing distrust in authorities and opposition to mandatory vaccination	“We won’t be blackmailed by those in charge”. Male, ≥25 years old. “Not trying to profit from the vaccine or have politicians push for mandatory vaccinations”. Female, 21–22 years old, Health science “I might have received it [the vaccine] if they hadn’t pressured me so much”. Female, 23–24 years old, Management and Economics science. “If it was not mandatory” Female, 21–22 years old, Health science “The ability to choose freely, based on complete information”. Female, ≥25 years old, Agricultural science

## Data Availability

The data that support the findings of this study are available from the corresponding author upon reasonable request.

## Supplementary Materials

The following supporting information can be downloaded at https://www.mdpi.com/article/10.3390/healthcare13101115/s1, Study questionnaire.
