# Supplementary material for: Factors Influencing COVID-19 Vaccination Hesitancy and Booster Dose Adherence Among University Students: A Cross-Sectional Study in Crete, Greece
Source: Healthcare (Basel). 2025 May 11;13(10):1115. doi: 10.3390/healthcare13101115 (PMC12110812; doi:10.3390/healthcare13101115)
Supplement: Supplementary file 1 [file healthcare-13-01115-s001.zip › healthcare-3597081-supplementary.pdf]

ATTITUDES AND PERCEPTIONS ABOUT THE VACCINE AGAINST THE COVID-19 CORONAVIRUS IN HMU STUDENTS

1. **Place of residence:**
2. **Sex:** Male □ Female □
3. **Age:**
4. **Smoking:** Yes □ Not □ Ex□
5. **School of study:**
6. **Course of study:**
7. **Year of study:**
8. **Marital status:**
9. **General health condition:** good □ moderate □ Bad □
10. **Chronic Diseases:**
11. **Medication:**
12. **Do you belong to a vulnerable group?** Yes No
13. **Do people who live with you belong to a vulnerable group?** Yes No
14. **COVID vaccination:**

    Yes No

    When; Number of installments:

    Side effects? Mild □ Moderate □ Severe □
15. **COVID-19 illness:**

    When;

    How would you characterize your illness? Mild Moderate Severe

16. Why would you take the Covid-19 vaccine?

1. My own initiative
2. Informing my doctor
3. Fear of serious illness
4. Media briefing
5. Due to profession
6. Free Provision
7. Other reasons

17. Why would you not get the Covid-19 vaccine?

1. Incomplete information
2. Fear of side effects
3. Reported side effects in family/social environment
4. It is part of a conspiracy
5. Doubt efficacy (does not protect 100%)
6. It has not been adequately tested
7. I've had other vaccines that protect me
8. Due to pregnancy
9. I've got COVID-19, I don't need it
10. I prefer to get sick
11. I believe that I will not get sick or that whatever I get sick will not be serious

12. I follow my family's point of view

13. Commercial reasons promote the vaccine

14. I don't believe in vaccination in general

15. Other reasons

18. It influences your opinion on whether to get the Covid-19 vaccine:

1. Religion

2. Political leadership

3. Scientists' opinion

4. The media and the internet

5. The anti-vaccination movement

19. Are you worried about getting infected with COVID-19?

Yes No

20. Would You - Have you had the flu vaccine?

Yes No
